# Antibiotic Resistance of *Campylobacter* Species in a Pediatric Cohort Study

**DOI:** 10.1128/AAC.01911-18

**Published:** 2019-01-29

**Authors:** Francesca Schiaffino, Josh M. Colston, Maribel Paredes-Olortegui, Ruthly François, Nora Pisanic, Rosa Burga, Pablo Peñataro-Yori, Margaret N. Kosek

**Affiliations:** a Department of International Health, Johns Hopkins Bloomberg School of Public Health, Baltimore, Maryland, USA; b Faculty of Science and Philosophy, Universidad Peruana Cayetano Heredia, Lima, Peru; c Asociación Benéfica Prisma, Iquitos, Loreto, Peru; d Department of Environmental Health and Engineering, Johns Hopkins Bloomberg School of Public Health, Baltimore, Maryland, USA; e U.S. Naval Medical Research Unit 6 (NAMRU-6), Iquitos, Loreto, Peru

**Keywords:** *Campylobacter*, Iquitos, MAL-ED, antibiotic resistance, diarrhea

## Abstract

The objective of this study was to determine the phenotypic patterns of antibiotic resistance and the epidemiology of drug-resistant *Campylobacter* spp. from a low-resource setting. A birth cohort of 303 patients was followed until 5 years of age. Stool samples from asymptomatic children (*n* = 10,008) and those with diarrhea (*n* = 3,175) were cultured for *Campylobacter*. Disk diffusion for ciprofloxacin (CIP), nalidixic acid (NAL), erythromycin (ERY), azithromycin (AZM), tetracycline (TE), gentamicin (GM), ampicillin (AMP), amoxicillin and clavulanic acid (AMC), ceftriaxone (CRO), chloramphenicol (C), and trimethoprim-sulfamethoxazole (TMS) was determined. Antibiotic resistances in Campylobacter jejuni and non-C. jejuni isolates from surveillance and diarrhea samples were compared, and the association between personal macrolide exposure and subsequent occurrence of a macrolide-resistant *Campylobacter* spp. was assessed. Of 917 *Campylobacter* isolates, 77.4% of C. jejuni isolates and 79.8% of non-C. jejuni isolates were resistant to ciprofloxacin, while 4.9% of C. jejuni isolates and 24.8% of non-C. jejuni isolates were not susceptible to azithromycin. Of the 303 children, 33.1% had been diagnosed with a *Campylobacter* strain nonsusceptible to both azithromycin and ciprofloxacin. Personal macrolide exposure did not affect the risk of macrolide-resistant *Campylobacter*. Amoxicillin and clavulanic acid (94.0%) was one of the antibiotics with the highest rates of susceptibility. There is a high incidence of quinolone- and macrolide-resistant *Campylobacter* infections in infants under 24 months of age. Given the lack of association between personal exposure to macrolides and a subsequent *Campylobacter* infection resistant to macrolides, there is a need to evaluate the source of multidrug-resistant (MDR) *Campylobacter*. This study provides compelling evidence to propose amoxicillin/clavulanic acid as a treatment for campylobacteriosis.

## Abstract (precis)

The objective of this study was to determine the phenotypic patterns of antibiotic resistance and the epidemiology of drug-resistant *Campylobacter* spp. from a low-resource setting.

## INTRODUCTION

*Campylobacter* is a globally disseminated Gram-negative zoonotic bacterium that is the main cause of gastrointestinal disease in children and adults. The principal transmission pathways of *Campylobacter* include fecal contamination of undercooked meat, water, and other poultry by-products (1). In industrialized nations, Campylobacter jejuni is the most common species of *Campylobacter* isolated in patients with diarrheal disease, followed by Campylobacter coli (2–5). In low-income settings, the epidemiology of campylobacteriosis may be distinct, and recent studies suggest that other *Campylobacter* species such as C. hyointestinalis and C. concisus are a major cause of diarrheal disease in pediatric populations were the disease is endemic (6–8).

In recent years, C. jejuni and C. coli resistance to antibiotics has increased throughout the world (9–12). Specifically, high levels of resistance to fluoroquinolones and macrolides in C. jejuni and C. coli isolates, as well as emerging resistance to aminoglycosides, have been reported in human and animal isolates (11–14). Although *Campylobacter*-associated diarrhea is generally a self-limiting disease and antibiotic treatment is not commonly advised (15), for patients with severe symptoms, dysentery, and compromised immunological systems and pregnant women, treatment is warranted (16). Fluoroquinolones, specifically, ciprofloxacin, were once considered the main treatment option (16). However, due to high levels of resistance to this drug, macrolides, namely, azithromycin, are the currently recommended first line of treatment for regular citizens and deployed military personnel as stated by the Infectious Disease Society of America, American College of Gastroenterology, and the U.S. military (16–21). Nonetheless, the recent emergence of macrolide resistance is beginning to threaten this treatment option (11, 22, 23).

Published statistics on antibiotic resistance in *Campylobacter* isolates are limited by the number of isolates analyzed. The vast majority of data comes from studies involving a few dozen isolates, and only a few studies report data on more than one hundred isolates, which includes data from countries such as Thailand (24, 25), South Korea (26), China (27), Japan (28), Tanzania (29), Peru (12, 30), Sweden (31, 32), Finland (33), and Poland (34, 35).

Two global multicenter studies identified *Campylobacter* as a pathogen with one of the highest attributable burdens of pathogenic diarrhea among children under 2 or 5 years of age (36, 37). Specifically, the results from the Etiology, Risk Factors, and Interactions of Enteric Infections and Malnutrition and the Consequences for Child Health (MAL-ED) cohort in Iquitos, Peru, show that the highest incidence of diarrhea in children under 12 months of age is attributed to *Campylobacter* infections. Among children under 2 years of age, *Campylobacter* is the second leading diarrhea-causing pathogen (36). In this same setting in a separate cohort of children 0 to 5 years of age, symptomatic and asymptomatic *Campylobacter* infections were associated with reduced weight over a 3-month period, and severe symptomatic infections were associated with reduced linear growth (38). A reanalysis of the Global Enteric Multicenter Study (GEMS) case-control study found *Campylobacter* among the top 6 pathogens with the highest attributable burden to diarrhea. Thus, there is compelling evidence of disease burden attributable to *Campylobacter* infections in pediatric populations of low-resource settings. However, neither of these studies have reported (to date) antibiotic resistance patterns of the *Campylobacter* isolates obtained.

The majority of human observational studies reporting antibiotic resistance in *Campylobacter* have been conducted in high-income settings, and only a limited number of longitudinal studies have assessed antibiotic resistance in *Campylobacter* species isolates from children. Therefore, there is a need to present evidence on the burden of antibiotic-resistant *Campylobacter* on pediatric populations. Characterizing the patterns and epidemiology of antibiotic-resistant *Campylobacter* in a low-resource tropical area is also of critical importance for guiding clinical management, as routine antimicrobial resistance (AMR) testing is not done in most settings where the disease is endemic, as well as for guiding clinical antibiotic stewardship and regulating veterinary antibiotic usage in both low- and high-income settings. As 18% of culture-confirmed cases of *Campylobacter* within the United States are associated with international travel, the importance of characterizing drug-resistant *Campylobacter* infections in developing areas of the world (39) is relevant to U.S. and European populations as well as the populations from which the data are derived. Finally, we evaluate the effect of azithromycin and erythromycin administration for therapeutic purposes on the risk of acquisition of macrolide-resistant *Campylobacter* strains.

## RESULTS

A total of 303 children were enrolled between 2009 and 2012 and followed until 5 years of age. From these, 242 children (79.9%) had tested positive for *Campylobacter* spp. by culture (8). Between March 2010 and February 2016, 10,008 surveillance fecal samples, 3,174 diarrhea samples, and 22 samples of undetermined status were submitted for stool culture (*N* = 13,204). Nine-hundred seventeen *Campylobacter* species isolates were cultured: 664 were from surveillance (asymptomatic) samples and 252 were from diarrheal samples, translating into an isolation rate of 6.6% among surveillance fecal samples and 7.9% among diarrhea samples. One *Campylobacter* strain was isolated from a sample of undetermined status. C. jejuni was identified in 596 samples (65.0%), of which 169 (28.4.0%) correspond to diarrheal samples. Non-C. jejuni isolates were identified in 321 samples (35.0%), of which 83 (25.9%) corresponded to diarrheal samples (Table 1). Throughout the follow-up period, 26.6% of children had only 1 isolate of *Campylobacter* cultured, and 53.3% of children had between 2 and 4 isolates identified.

**TABLE 1:** Types of fecal samples associated with *Campylobacter* species

Species^*a*^	% (*n*) of samples		Total (*n*)
	Diarrhea	Surveillance	
C. jejuni	28.4 (169)	71.5 (426)	596^*b*^
*Non*-C. jejuni	25.9 (83)	74.1 (238)	321

a In 917 stool samples from which *Campylobacter* was isolated, non-*jejuni* isolates accounted for 35% of the total number of *Campylobacter* isolates.

b One C. jejuni isolate was not determined as diarrhea or surveillance.

**FIG 1:**
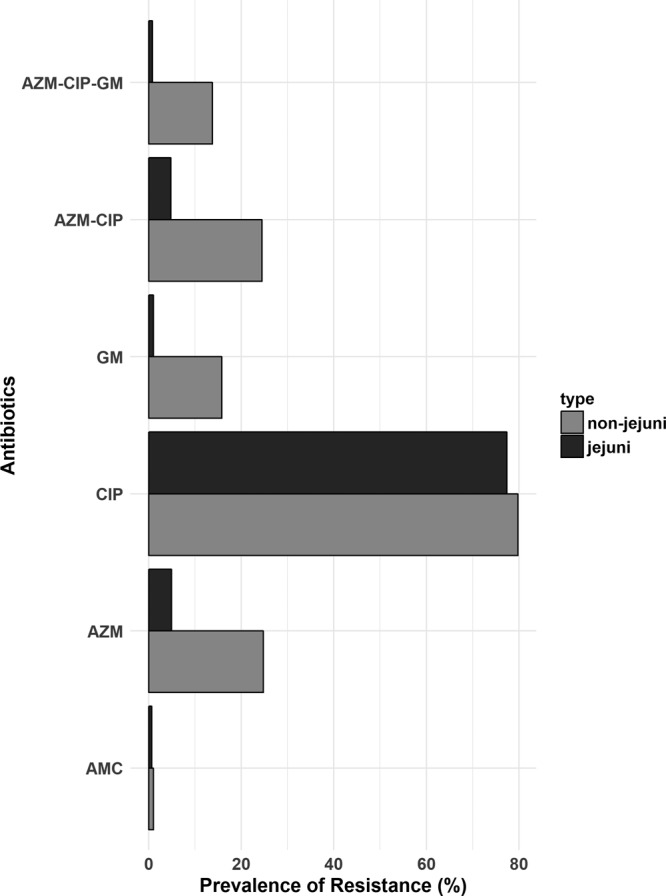
Azithromycin, gentamicin, ciprofloxacin, and amoxicillin-clavulanic acid phenotypic multidrug resistance in *Campylobacter* spp.

The prevalence of phenotypic resistance for all antibiotics tested is presented in Table 2. The most effective oral antibiotic was amoxicillin and clavulanic acid. The highest levels of resistance were recorded for ciprofloxacin: 77.4% of C. jejuni isolates and 79.8% of non-C. jejuni isolates. Azithromycin resistance was detected in only 4.9% of C. jejuni isolates and in 24.8% of non-C. jejuni isolates. All azithromycin-resistant isolates were also ciprofloxacin resistant with the exception of 1 C. jejuni and 1 non-C. jejuni isolate. Other striking patterns include tetracycline resistance in 55.8% of C. jejuni isolates and in 49.0% of non-C. jejuni isolates, as well as gentamicin resistance in 15.8% of non-C. jejuni isolates. No significant longitudinal trends in antibiotic resistance and multidrug resistance were observed throughout the 5-year study period. Multidrug resistance (defined as phenotypic nonsusceptibility to 3 or more classes of antibiotics) was observed in 56.8% (335/590) of C. jejuni isolates and 59.1% (176/298) of non-C. jejuni isolates. Concomitant phenotypic resistance to ciprofloxacin and azithromycin was observed in 24.5% (75/298) of non-C. jejuni isolates and in 4.8% (28/588) of C. jejuni isolates. Concomitant resistance to ciprofloxacin, azithromycin, and gentamicin was observed in 13.8% (41/298) of non-C. jejuni isolates and in 0.8% (5/596) of C. jejuni isolates (Fig. 1).

**TABLE 2:** Phenotypic antibiotic susceptibility of Campylobacter jejuni and non-Campylobacter jejuni isolates

Antibiotic	C. jejuni isolates				Non-C. jejuni isolates			
	% (*n*)^*a*^			Total (*N*)	% (*n*)			Total (*N*)
	R	I	S		R	I	S	
CIP	77.4 (455)	1.7 (10)	20.9 (123)	588	79.8 (237)	1.1 (3)	19.2 (57)	297
NAL	64.9 (383)		35.1 (207)	590	80.1 (237)		19.9 (59)	296
ERY	5.3 (31)	0.2 (1)	94.6 (556)	588	25.2 (75)		74.8 (223)	298
AZM	4.9 (29)	0.2 (1)	94.9 (558)	588	24.8 (74)		75.2 (224)	298
TE	55.8 (328)		44.2 (250)	588	49 (146)		51 (152)	298
AMP	46.8 (276)	3.9 (23)	49.3 (291)	590	50.7 (151)	8.7 (26)	40.6 (121)	298
AMC	0.7 (4)	0.3 (2)	98.9 (584)	590	1 (3)	5.0 (15)	94.0 (280)	298
C	0.2 (1)		99.8 (589)	590	0.3 (1)	0.3 (1)	99.3 (296)	298
CRO	44.7 (263)	28.7 (169)	26.7 (157)	589	55.0 (164)	22.1 (66)	22.8 (68)	298
GM	1 (6)	0.2 (1)	98.8 (581)	589	15.8 (47)	0.3 (1)	83.9 (250)	298
TMS	85.2 (501)	1.7 (10)	13.1 (77)	588	80.9 (241)	3.4 (10)	15.8 (47)	298

a R, resistant; I, intermediate; S, susceptible.

Of the 303 children enrolled and followed up, a total of 80 (33.1%) were found to have a *Campylobacter* strain nonsusceptible to both azithromycin and ciprofloxacin. Additionally, 26 (8.6%) had a diarrhea episode due to an azithromycin-resistant isolate, of which all strains where also resistant to ciprofloxacin, with the exception of one. Ciprofloxacin resistance was far more common than azithromycin resistance: 232 (76.6%) children ever diagnosed with a resistant ciprofloxacin isolate and 41.6% of children had a diarrhea episode due to a ciprofloxacin-resistant *Campylobacter* spp.

The mean age at which a child presented with the first diarrhea episode due to a ciprofloxacin-resistant isolate was 12 months, and the mean age was 18 months for the first diarrhea episode due to an azithromycin-resistant isolate (*P* < 0.001). Among the 29 diarrhea episodes caused by azithromycin-nonsusceptible isolates, 10 were diagnosed during the 12 months, and all but two were diagnosed before the child was 36 months of age. Within the first 12 months of life, 57 (18.8%) children had already experienced diarrhea caused by a ciprofloxacin-resistant isolate. This increased to 107 (35.3%) by 24 months and to 122 (39.9%) by 36 months.

Of the 303 children, on average, a child was on antibiotics for 5.6% of the days during their first 5 years of life, of which, macrolides (azithromycin or erythromycin) account for 1.1% of this time period. The median number of courses of macrolides a child received was 3 (interquartile range [IQR], 1 to 5 courses), which translates to an average of 12 days (IQR, 4 to 21 days). A child’s risk of being culture positive for a macrolide-resistant *Campylobacter* was not statistically different between children with high macrolide exposure and those with low macrolide exposure. Similar results were found for the time to first isolation of a macrolide-resistant *Campylobacter*. The cumulative effect of macrolide intake early in life was not statistically associated with the odds of acquisition of a macrolide-resistant isolate.

## DISCUSSION

Of 917 *Campylobacter* isolates obtained from children enrolled in the MAL-ED cohort of Peru, amoxicillin and clavulanic acid was the antibiotic with the highest rate of susceptibility. Resistance to ciprofloxacin was expressed in 77.4% of C. jejuni isolates and in 79.8% of non-C. jejuni isolates, while resistance to azithromycin was found in 4.9% of C. jejuni isolates and in 24.8% of non-C. jejuni isolates.

Previous studies assessing antibiotic resistance in human gastroenteritis associated with *Campylobacter* have found various levels of resistance. Studies in the United Kingdom, South Korea, Israel, and Peru have demonstrated lower levels of fluoroquinolone resistance, ranging from 24% to 65% (2, 12, 26, 40). However, observational studies from China (87%) and Japan (90%) report higher estimates of ciprofloxacin resistance (41, 42). Data from the National Antimicrobial Resistance Monitoring System (NARMS) of the United States shows that in 2015, 25.3% of C. jejuni isolates and 39.8% of C. coli isolates were resistant to ciprofloxacin. Macrolide resistance is even more wide ranging. We report an overall resistance of 24.8% for non-C. jejuni isolates, a higher prevalence than was reported in a nearby region in 2010 (10.0%) (12). However, the C. jejuni resistance to macrolides (4.9%) in our study is lower than what was previously reported in the same region (14.9%) (12). Worldwide, macrolide resistance in human *Campylobacter* species isolates varies between 0.8% in South Korea, 2.2% in the United Kingdom, 12.5% in Thailand, 21.8% in China, and 22.2% in India (10, 26, 40–42). Additionally, cases of *Campylobacter*-associated travelers’ diarrhea in U.S. military troops show as low as 2% nonsusceptibility to azithromycin (43). NARMS data showed azithromycin resistance in 2.7% of C. jejuni isolates and in 12.7% of C. coli isolates. According to the 2015 NARMS report, between 2011 and 2015, erythromycin resistance in human-associated C. coli isolates increased from 2.7% to 12.7%.

Antibiotic resistance of *Campylobacter* isolates from animal sources, most importantly, poultry, also appear to be country dependent. A study conducted in China reported that 73.2% of C. coli isolates were erythromycin resistant, and a study from Spain similarly reported 73.0% resistance (44, 45). Reports from Latin America are limited. Sierra-Arguello et al. reported an overall 2% resistance to erythromycin (46). Given that azithromycin is the currently prescribed treatment for campylobacteriosis (16, 19, 21), our reported levels of resistance to macrolides in a pediatric population is worrisome but not unique.

The rise in fluoroquinolone and macrolide resistance has been attributed to antibiotic administration for growth promotion in poultry and hog production (9, 47, 48). However, *Campylobacter* antibiotic resistance in animal hosts has not been well assessed in this region. Poultry production and commercialization in Iquitos includes market vendors from concentrated animal operation facilities as well as from small backyard production within households. Slaughtering is generally performed at home or within the live markets, yet there is little evidence that characterizes the risk of *Campylobacter* contamination and infection within the poultry industry in this region. The use of antibiotics as growth promoters is not regulated in Peru, and over-the-counter access to antibiotics from local vendors is common. Therefore, further studies that characterize *Campylobacter* antibiotic resistance patterns in poultry and other animal hosts are required to evaluate the zoonotic component of *Campylobacter* epidemiology in this area. An incredibly common infection could potentially end up untreatable if effective control interventions involving antibiotic stewardship in both humans and animals are not promptly executed.

Another concern regarding the acquisition of widespread drug resistance is the recent reports of standard periodic dosing in early childhood to decrease mortality (49). The apparent volume of distribution is very large, 25 to 30 liter/kg, and slow release from intracellular compartments extends the time for which the drug is available at subinhibitory concentrations (50). The agent is principally excreted unchanged in the feces, with an elimination half-life of 2 to 4 days. Prolonged subtherapeutic concentrations and the active agent elimination route into the gastrointestinal tract both favor the selection of drug resistance to a greater extent than the more routine use of antibiotics. Our analysis shows that intermittent azithromycin use for therapeutic purposes is unlikely to have a population-level effect on the emergence of *Campylobacter* resistance to macrolides. Therefore, we stress the need to explore macrolide use for animal production purposes and its effect on the emergence of *Campylobacter* resistance. The loss of azithromycin as a widespread feasible option for the treatment of campylobacteriosis, as is happening here, has meaningful consequences given the prevalence of both symptomatic disease and enteropathy associated with this infection.

Gentamicin resistance is a novel phenomenon in *Campylobacter* isolates (14). We report an overall prevalence of 15.8% for non-C. jejuni isolates and 1.0% resistance for C. jejuni isolates. This is a lower estimate than the 28.8% reported in human diarrheal samples from China yet higher than reports from South Korea (6.6%) (26, 42). In Belgium, gentamicin resistance in poultry isolates has increased from almost being nonexistent in 2004 to approximately 20% by 2009 (11). Multiple phosphotransferase (*aph*) genes are commonly associated with aminoglycoside resistance, and many of them have been identified as transferable (13, 14, 51).

Multidrug resistance (MDR) was observed in 56.8% (335/590) of C. jejuni isolates and 59.1% (176/298) of non-C. jejuni isolates. This is striking given that the only previous evidence of such higher rates came from poultry isolates in China, where 81.1% of C. jejuni isolates and 47.7% of C. coli isolates were resistant to 3 or more antibiotics (44). It is important to consider the definition of MDR when comparing overall susceptibility trends across the globe. Whole-genome sequencing explorations have identified a transferable multidrug resistance genomic island (MDRGI) which contains antibiotic resistance determinants for quinolone and macrolides, as well as tetracycline and aminoglycosides (52). Thus, future studies exploring molecular resistance determinants in this species should aim to identify the prevalence of this MDRGI. Clinically relevant MDR can be considered to be toward both ciprofloxacin and azithromycin. This was observed in 4.8% of C. jejuni and 24.5% of non-C. jejuni isolates, a highly significant percentage considering that this study was conducted in a pediatric population in a remote tropical setting.

Surprisingly, amoxicillin and clavulanic acid expressed the lowest proportion of resistance, although this has been noted in earlier studies from Spain (53). Therefore, we propose that this antibiotic should be considered a treatment option for *Campylobacter*-associated gastroenteritis when isolates are nonsusceptible against fluoroquinolones and macrolides.

We acknowledge that disc diffusion breakpoints for antibiotics other than erythromycin, ciprofloxacin, and tetracycline have not been standardized for *Campylobacter*. More commonly utilized are the MIC breakpoints; yet, these were not used in this study. Second, we did not explore the presence and diversity of antibiotic resistance genes in these samples. Quinolone resistance has been attributed to target mutations in the quinolone resistance-determining region (QRDR) (54–56), as well as to the presence of the cmeABC efflux pump conferring intrinsic resistance to fluoroquinolones (57–59). The reported mechanisms of macrolide resistance include target mutations of the 23S rRNA genes (23, 55, 60), target mutations in ribosomal proteins (55, 61), and ribosomal methylation encoded by the *erm* genes, of which the *erm*(B) gene is associated with high-level resistance, mostly in C. coli (23, 55, 62, 63). In Peru, a *gyrA* mutation associated with quinolone resistance and a 23S rRNA mutation associated with macrolide resistance were detected within a limited sample of *Campylobacter* isolates from a pediatric cohort in Lima (30, 64).

A large proportion of diarrhea episodes associated with resistant *Campylobacter* against current treatment recommendations indicates that this multidrug-resistant campylobacteriosis is highly endemic in the Peruvian Amazon. To our knowledge, no other cohort studies report the susceptibility patterns of *Campylobacter* infections in a pediatric population in low-resource settings, and this is one of the few studies available reporting statistics on *Campylobacter* antibiotic resistance in more than 800 isolates cultured from children. Future studies that evaluate the molecular determinants of antibiotic resistance in thoroughly characterized *Campylobacter* species isolates will shed light on the origins and dynamics of this infection in the Peruvian Amazon.

### Conclusions.

*Campylobacter* isolates from children under 5 years of age in the Peruvian Amazon show a higher prevalence of phenotypic resistance than other regional and country estimates. As expected, non-C. jejuni isolates showed higher levels of azithromycin resistance than C. jejuni isolates. Although both C. jejuni and non-C. jejuni isolates showed a high prevalence of ciprofloxacin resistance, this phenotypic trait is more common among C. jejuni isolates. We report for the first time in the region moderate to increased levels of gentamicin resistance, especially among non-C. jejuni isolates. When considering treatment options for gastroenteritis associated with *Campylobacter*, amoxicillin and clavulanic acid should be considered.

## MATERIALS AND METHODS

### Setting and study design.

The MAL-ED study is a multisite, prospective, community-based cohort study. Between 2009 and 2012, 303 newborns were enrolled within the first 17 days of life and were followed up until 5 years of age. The study site in Peru consisted of 3 communities 15 km southeast of Iquitos, the largest city of the Peruvian Amazon. Details of enrollment and surveillance procedures have been published previously (65). Surveillance stool samples were collected monthly, and diarrheal samples were collected within 48 h of a reported episode. A diarrheal episode was defined as three or more loose stools within 24 h or one dysenteric stool. Subjects were visited 2 times weekly and a continual symptom history was recorded, which included data on antibiotic use, generating a continual daily history for the period of study. Further data and sample collection procedures have also been described previously (66).

### Laboratory procedures.

Diarrheal and surveillance fecal samples were placed in Cary Blair transport medium and were processed within 12 h (8). Stools were inoculated on *Campylobacter* agar base supplemented with Blaser’s supplement (Becton Dickinson, Sparks, MD) containing vancomycin, cephalothin, trimethoprim, polymyxin, and amphotericin B. Plates were incubated for 48 h at 42°C at 5% O_2_, 10% CO_2_, and 85% N_2_. If no growth was observed, agar plates were held at least 72 h to confirm this finding. Gram-negative colonies demonstrating typical *Campylobacter* morphology were assessed using oxidase and catalase tests as well as Gram staining. Colonies with typical *Campylobacter* species morphology as well as oxidase and catalase activity were further assessed using the hippurate hydrolysis test to distinguish Campylobacter jejuni from non-C. jejuni (38). *Campylobacter* species were identified as C. jejuni if positive for hippurate hydrolysis and non-C. jejuni if negative for hippurate hydrolysis. We referred to isolates as C. jejuni or non-C. jejuni throughout the paper due to uncertainty as to whether all non-*jejuni Campylobacter* truly represent C. coli.

Phenotypic antimicrobial susceptibility patterns were assessed using standard disc diffusion (Kirby-Bauer) methods. Resistance to the following antibiotics was tested: ciprofloxacin (CIP), nalidixic acid (NAL), erythromycin (ERY), azithromycin (AZM), tetracycline (TE), gentamicin (GM), ampicillin (AMP), amoxicillin and clavulanic acid (AMC), ceftriaxone (CRO), chloramphenicol (C), and trimethoprim-sulfamethoxazole (TMS). Zone diameter breakpoints (millimeters) for *Campylobacter* spp. validated by the Clinical and Laboratory Standards Institute (CLSI document M45) were applied to assess ciprofloxacin, erythromycin, azithromycin, and tetracycline resistance. CLSI zone diameter breakpoints (millimeters) for Enterobacteriaceae were used for the remaining antibiotics for which there are no established breakpoints for *Campylobacter* spp. Table S1 in the supplemental material displays all zone diameter breakpoints.

### Data management and analysis.

CLSI standards were used to categorize isolates as susceptible, resistant, or intermediate. The proportion nonsusceptible to each antibiotic was tabulated for both C. jejuni and non-C. jejuni isolates when resistant and intermediate categories were combined. Multidrug resistance (MDR) was defined as an isolate expressing phenotypic nonsusceptibility to three or more classes of antibiotics (67). Cephalosporins (CRO) were not included in this classification given the intrinsic resistance of *Campylobacter* spp. Pearson’s chi-square was used to test the differences in resistance to all antibiotics between C. jejuni and non-C. jejuni isolates, as well as between surveillance and diarrhea samples.

Macrolide exposure was analyzed as continuous time-varied exposure as well as binary exposure. High macrolide exposure was defined as higher than the population’s median number of courses of antibiotics (68). Risk ratios for the association between daily macrolide (AZM and ERY) exposure and the isolation of macrolide-resistant *Campylobacter* were modeled using log-binomial regression fitted with generalized linear models and robust variance estimation. Differences in the times to first detection of a macrolide-resistant *Campylobacter* isolate between those with high and low macrolide exposure were assessed by comparing cumulative incidence curves and performing log-rank tests of statistical significance. Finally, the effects of the cumulative exposure of macrolide use early in life (number of days with macrolide intake before 6 months and 12 months of age) and the odds of acquiring a macrolide-resistant *Campylobacter* later in life (after 12 and 18 months of age) were assessed by fitting logistic regression models. Data manipulation and statistical analysis were performed with STATA 14 (Stata Corp., College Station, TX) and R (version 3.3.2).

## Supplementary Material

Supplemental file 1
