# Supplementary material for: Antibiotic Resistance of Campylobacter Species in a Pediatric Cohort Study
Source: Antimicrob Agents Chemother. 2019 Jan 29;63(2):e01911-18. doi: 10.1128/AAC.01911-18 (PMC6355604; doi:10.1128/AAC.01911-18)
Supplement: Supplemental file 1 [file 392e6918224c7179d350ed08c9344ea4_AAC.01911-18-s0001.pdf]

Supplementary Table 1. Disk diameters used to classify isolates as sensitive, intermediate or resistant.

Antimicrobial Class and Agent	Abbreviation	Resistant (mm)	Intermediate (mm)	Susceptible (mm)	Source
**Quinolones**					
Ciprofloxacin	CIP	≤20	20-24	≥24	1
Nalidixic Acid	NAL	≤19	-	≥20	2
**Macrolides**					
Erythromycin/ Azithromycin	ERY	≤12	13-15	≥16	1
**Tetracycline**					
Tetracycline	TET	≤22	23-25	≥26	1
**Aminoglycoside**					
Gentamicin	GEN	≤12	13-14	≥15	3
**Beta-Lactams**					
Ampicillin	AMP	≤8	10-15	≥15	3
Amoxicillin + Clavulanic Acid	AMC	≤13	14-17	≥18	3
Ceftriaxone	CRO	≤19	20-22	≥23	3
**Others**					
Trimethoprim-sulphamethoxazole	TMS	≤10	11-15	≥16	3
Chloramphenicol	C	≤12	13-17	≥18	3

Source:

1. CLSI. Methods for Antimicrobial Dilution and Disk Susceptibility Testing of Infrequently Isolated or Fastidious Bacteria. Rd ed. CLSI guideline M45. Wayne, PA: Clinical and Laboratory Standards Institute; 2016
2. BSAC. British Society for Antimicrobial Chemotherapy. Methods for Antimicrobial Susceptibility Testing. 2013
3. CLSI. Performance Standards for Antimicrobial Susceptibility Testing. 27th ed. CLSI supplement M100. Wayne, PA: Clinical and Laboratory Standards Institute; 2016 Susceptibility Testing of Infrequently Isolated or Fastidious Bacteria, 3rd Edition
